# Habituation and sensitization in primary headaches

**DOI:** 10.1186/1129-2377-14-65

**Published:** 2013-07-30

**Authors:** Gianluca Coppola, Cherubino Di Lorenzo, Jean Schoenen, Francesco Pierelli

**Affiliations:** 1 Department of Neurophysiology of Vision and Neurophthalmology, G.B. Bietti Foundation IRCCS, Via Livenza 3, 00198, Rome, Italy; 2 Don Carlo Gnocchi Onlus Foundation, Milan, Italy; 3 Headache Research Unit, University Department of Neurology & GIGA-Neurosciences, Liège University, Liège, Belgium; 4 IRCCS Neuromed, Pozzilli (IS), Italy

**Keywords:** Migraine, Tension-type headache, Cluster headache, Trigeminal autonomic cephalalgias, Sensitization, Habituation, Evoked potentials, Reflex, Pain mechanisms, Thalamocortical dysrhythmia

## Abstract

The phenomena of habituation and sensitization are considered most useful for studying the neuronal substrates of information processing in the CNS. Both were studied in primary headaches, that are functional disorders of the brain characterized by an abnormal responsivity to any kind of incoming innocuous or painful stimuli and it’s cycling pattern over time (interictal, pre-ictal, ictal). The present review summarizes available data on stimulus responsivity in primary headaches obtained with clinical neurophysiology. In migraine, the majority of electrophysiological studies between attacks have shown that, for a number of different sensory modalities, the brain is characterised by a lack of habituation of evoked responses to repeated stimuli. This abnormal processing of the incoming information reaches its maximum a few days before the beginning of an attack, and normalizes during the attack, at a time when sensitization may also manifest itself. An abnormal rhythmic activity between thalamus and cortex, namely thalamocortical dysrhythmia, may be the pathophysiological mechanism subtending abnormal information processing in migraine. In tension-type headache (TTH), only few signs of deficient habituation were observed only in subgroups of patients. By contrast, using grand-average responses indirect evidence for sensitization has been found in chronic TTH with increased nociceptive specific reflexes and evoked potentials. Generalized increased sensitivity to pain (lower thresholds and increased pain rating) and a dysfunction in supraspinal descending pain control systems may contribute to the development and/or maintenance of central sensitization in chronic TTH. Cluster headache patients are chrarcterized during the bout and on the headache side by a pronounced lack of habituation of the brainstem blink reflex and a general sensitization of pain processing. A better insight into the nature of these ictal/interictal electrophysiological dysfunctions in primary headaches paves the way for novel therapeutic targets and may allow a better understanding of the mode of action of available therapies.

## Review

### Introduction

Among the general population, headaches are highly prevalent and receive growing attention not only because they affect people’s quality of life, but also because they have a significant economic impact. Idiopathic or primary headache syndromes are disorders in which there is a temporary or permanent dysfunction of the central nervous system, often genetically determined, without apparent organic lesion. They include migraine, tension headache, and the trigeminal autonomic cephalalgias among which "cluster headache". Progress in headache research has benefited from the International Classification of Headache Disorders (ICHD), and its revisions [1,2], because they have provided operational diagnostic criteria allowing for a better comparison of clinical data between headache centres.

In the recent publication of the Global Burden of Disease survey 2010, tension-type headache and migraine are the second and third most prevalent disorders in the world, and migraine is recognized as the seventh highest cause of disability in the world [3]. The large and still growing scientific knowledge on their pathophysiological mechanisms has contributed the recognition of headaches as neurological conditions worth of interest. In fact, although scientists have not completely disentangled the complicated puzzle of primary headache pathophysiology, great advances were made during the last 2 decades with the help of the new research armamentarium.

Clinical neurophysiology methods, in particular, have allowed in vivo measurements of the headache patients’ electrocortical responses to various sensory stimuli. They are atraumatic, non-invasive and complementary to modern neuroimaging techniques, and thus suitable to study functional disorders as primary headaches.

In their episodic forms, primary headaches are defined as paroxysms (the attacks) separated by remissions of variable lengths. Several studies focused therefore for ictal versus interictal electrophysiological abnormalities, in order to understand the predisposition and the recurrent character of attacks. In this respect, among the various primary headaches, migraine is doubtless the best-studied headache type.

Migraineurs are characterized interictally by a generally increased sensitivity to visual (sensitivity to light), auditory (to sound), or somatic stimuli (allodynia) not only during the attack, but also outside of the attack. Researchers, trying to explain this phenomenon, observed that interictally migraineurs with and without aura show a time dependent amplitude increase of scalp-evoked potentials to repeated stereotyped stimuli with respect to normal subjects. This phenomenon was called “deficient habituation” and was only seen during the pain-free period for almost all sensory modalities. However, this is not a static phenomenon, but, as shown in several studies, habituation changes with the proximity to an attack, during the attack and when episodic migraine evolves to chronic migraine, a complication of migraine where sensitization, the opposite of habituation, makes its appearance, changing fundamentally the response pattern.

The present article, after providing an overview of the general concept of habituation and its opposite “sensitization”, will review the studies on habituation/sensitization in migraine and other primary headaches, performed with different clinical neurophysiology methods and emphasize in particular the more recent data.

### General concept

Habituation is defined as “a response decrement as a result of repeated stimulation” [4] and is a common feature of responses to any kind of sensory stimulation. It is an ubiquitous phenomenon observed in different experimental settings and in neuronal circuits of a wide range of complexity, from the withdrawal reflex of the gill and siphon in Aplysia to the autonomic and behavioral component of the whole-of-body reflex called the “orienting response” in humans [5-7].

Habituation is a multifactorial event of which the accompanying synaptic plastic mechanisms are still not totally elucidated. Several theories, or at least hypotheses, have been proposed over the years to explain this phenomenon [8]. In the 70's, Groves and Thompson proposed the “dual-process” theory, stating that two separate and opposing processes, depression (habituation) and facilitation (sensitization), compete to determine the final behavioural outcome after a sequence of repetitive stimuli [6]. According to the dual-process theory, sensitization is the side of the pendulum that, when present, prevails at the beginning of the stimulus session and accounts for the initial transitory increase in response amplitude, whereas habituation occurs later during the course of the recording session and accounts for the delayed response decrement [6]. At the synaptic level, the stimulus–response pathway interacts with an external “state” system represented by various “tonic” non-specific and motivational circuits, including the ascending reticular activating system and related structures. In humans, these structures comprise the monoaminergic nuclei in the brainstem, that are critically involved in the central processing of arousal, control of the signal-to-noise ratio generated by sensory stimuli at cortical and thalamic levels, and endogenous antinociception [9].

To avoid semantic misunderstanding, it should be noted that a response dishabituation does not refer to lack of habituation, but to a response sensitization or “heterosynaptic facilitation” as termed by Kandel and associates in their works on Aplysia [10]. In fact, dishabituation is an actual recovery of the habituated response caused by the interference of an unexpected stimulus markedly different from the habituating ones. Sensitization is an elementary form of behavioral plasticity, perhaps equal in importance to habituation and apparently generated by somewhat different neuronal mechanisms [11].

Habituation depends on a series of parametric properties or characteristics [5], which were revised and refined during a workshop in Vancouver in 2007 [12]. These characteristics dealt with both short- as well as long- term habituation and its opposite dishabituation.

The phenomenon of habituation is considered useful for studying the neuronal substrates of behavior, the mechanisms of learning processes, or information processing in the CNS both in health and in disease.

It must be noted that, during the last three decades, sensitization has assumed a wider meaning than habituation. Sensitization is not only considered a general behavioral response of augmentation to innocuous sensory stimuli, but it has also acquired a particular significance in the field of pain as an augmentation of sensory signalling in the central nervous system as a consequence of noxious stimulation (for a review see Woolf, 2011 [13]). The latter may be a peripheral injury activating small-fiber afferents that produce an increase in excitability, i.e. sensitize, nociceptive spinal cord neurons. This increase in excitability is responsible for the plastic changes in those neural structures belonging to the so-called “pain matrix”. It results in decreased nociceptive thresholds and increased responsiveness to noxious and innocuous peripheral stimuli, as well as expansion of the receptive fields of central nociceptors [14]. Overall, this transient or persistent state of higher reactivity is called “central sensitization”. Studies in the headaches field disclosed changes in pain sensitivity that were interpreted as reflecting central sensitization. A well-recognized clinical expression of central sensitization is cutaneous allodynia, which is prevalent during episodic migraine attacks [15,16] and in chronic migraine [17-19]. There is no common agreement yet about what causes and where starts the cascade of events that lead to central sensitization in migraine or in other primary headaches. However, some evidences point towards sequential sensitization of first-order or second-order trigeminovascular nociceptors via overt (aura) or possibly silent (without aura) cortical spreading depression waves or, more likely, via an indirect activation of pain modulatory structures in the brainstem (raphe magnus, locus coeruleus and other aminergic nuclei) and the forebrain (periaqueductal gray, rostroventral medulla) [20,21].

In human research, time-locked cortical potentials (EP) evoked by a sensory stimulation and reflex responses (RR) after electric stimulation of a peripheral nerve have been frequently used to study the habituation and sensitization phenomena. A common way of analysing these responses consists of either analysing single trials or averaging in blocks single epochs of EP or RR per subject, identifying the latencies and amplitudes or area-under-the-curves of the considered components, and using these parameters as a dependent variables in the statistical analysis. Data are usually expressed either in absolute or log-transformed values, and between single trials or blocks habituation is calculated either with linear regression (slope of the linear regression line) or with block ratios (change in amplitude expressed in percentage).

### Habituation in migraine

Neurophysiological data suggest that lack of habituation during stimulus repetition despite an initial normal or slightly lower response amplitude is a functional, probably genetically determined, property of the brain in migraineurs between attacks.

The very first study showing that habituation is decreased in patients affected by migraine without aura between attacks was carried out with contingent negative variation (CNV), a slow negative cortical response related to higher mental functions [22-26]. The CNV abnormality was more evident for the early than for the late component [24,27-33], and was assessed by visual [34,35] or auditory [36-38] oddball paradigms. The lack of habituation was confirmed several times by analyzing visual evoked potentials (VEP) in response to checkerboard pattern [39-49], and also by using magneto electroencephalography [50-52]. The abnormal visual information processing in migraine seems to be characterized by an initial response of normal or slightly lower amplitude followed by an amplitude increase, that, as stated above, corresponds neither to response sensitization nor to dishabituation, but to a lack of habituation [40-50]. The same phenomenon was also found with somatosensory [53-55] and auditory [56,57] evoked cortical potentials in migraine between attacks. Sand et al. (2008) recorded brainstem auditory evoked potentials (BAEPs) in migraine interictally and found lack of habituation in the wave IV-V [58], a datum that was recently confirmed also in a few migraineurs experiencing vertigo, especially when symptomatic [59]. Moreover, Sand et al. also observed a positive relationship between BAEP amplitudes and blood serotonin level in healthy controls, but not in migraine, a result that was interpreted as an evidence in support of the notion that a dysregulation of the serotonin system is linked to migraine pathogenesis [58].

Besides the innocuous evoked potentials, researchers have verified if the same habituation deficit might exist after noxious stimulation. The blink reflex (BR) obtained after supraorbital stimulation with a so-called “nociception-specific” electrode, a way to more specifically explore trigeminal nucleus caudalis activation, discloses an interictal habituation deficit during short [60,61] as well as long time courses [44,62]. Amplitude and habituation of another brainstem reflex, the click-evoked vestibulocollic reflex, was equally reduced in migraineurs between attacks compared with healthy subjects [63,64].

Brief radiant heat pulses, produced by CO2 laser stimulation, activate selectively Aδ and C fibres and generate an evoked potential that can be recorded from the temples (early component, N1) and the vertex (late components, N2-P2) of the skull. In episodic migraine the late component of laser evoked potentials (LEPs), mainly generated in the insular and anterior cingulate cortices, elicited by either cephalic (usually supraorbital) or extracephalic (usually hand dorsum) stimulation does not habituate during stimulus repetitions over short [65] or long durations [66-68]. Deficient habituation was also observed for the early N1 LEP component, mainly generated in the secondary somatosensory cortex [66,69]. In two studies performed in episodic migraine between attacks using contact-heat evoked potentials (CHEPS) and source localization with standardized LORETA (sLORETA) mapping it was shown that the lack of habituation to the noxious heat stimuli is probably related to the inability of the orbitofrontal cortex (OFC) to filter out correctly the pain information [70,71]. Since evidence from animal and human studies suggests a role for serotonin in the OFC-mediated descending inhibition of pain [72], the authors argue that a possible alteration of brain 5-HT neurotransmission may be responsible for this abnormal pain information processing in migraine, where decreased serotonergic disposition was previously reported [73-75],

Genetic load seems to play an important role in the mechanisms that produce physiologically altered habituation. In fact, the habituation deficit in migraineurs has a family character. This abnormality is present interictally not only in adults, but also in children in whom it is significantly correlated with that of their parents [28,76]. Moreover, asymptomatic subjects defined to be "at risk" for developing migraine, i.e. healthy but with first-degree relatives with migraine, present the same habituation deficit in evoked potentials and nociceptive blink reflex as established migraineurs, so that deficient habituation can be regarded as neurophysiological marker of pre-symptomatic migraine [29,62].

Since migraine is a recurrent paroxysmal disease characterized by attacks (ictal period) and variable pain-free periods (interictal), it was of interest to perform sequential recordings during the days preceding the attacks, immediately before or during the attack. During the days preceding the attack (pre-ictally) VEP and SSEP amplitudes increase and habituate normally [50,54,58,77,78], whereas CNV and P300 habituation is minimal and amplitude peaks [30,79,80], suggesting that, depending on sensory modality, the habituation deficit worsens interictally, reaches its maximum a few days before the attack and then normalizes during the attack.

Finally, it must be mentioned that among more than fifty positive studies, the habituation deficit in migraine during the pain-free phase was not confirmed in some studies [77,81-87]. It is not easy to explain why some research groups did not retrieve any habituation deficit in migrainous patients between attacks. For some Authors lack of blindness for diagnosis during the recording sessions may be an explanation [87], but the same research group did not find lack of habituation even with no blindness [77]. Another one could be the use of different patients’ selection criteria, such as recruitment of university students or medical staff instead of patients who spontaneously visited a Headache Clinic, the latter experiencing more day life discomfort from their migraine. Whatever the explanation, we must take into account that habituation deficit is not constant in migrainous patients. In fact, habituation degree may change not only interictally vs. pre-ictally vs. ictally, but also within the pain-free period with the distance since the last or next attack [88]. Moreover, specific genetics influence [76,89] and clinical fluctuations, such as spontaneous clinical worsening or improving of attacks frequency [90,91], may vary the baseline level of thalamocortical activation [92] and then the degree of habituation in migraine [55].

### Mechanisms of the habituation deficit in migraine

As mentioned above, the neural mechanisms underlying habituation remain poorly understood, and this uncertainty helps to explain why the abnormal habituation pattern in migraine still lacks a definitive consensual interpretation [93-95].

Neuromodulatory techniques, like repetitive transcranial magnetic stimulations (rTMS) and transcranial direct current stimulations (tDCS), were used to shed more light on the interictal abnormal information processing in migraine. In migraineurs activating high frequency rTMS over the visual or somatosensory cortices was able to increase for some minutes the amplitude of the first VEP and somatosensory evoked potentials (SSEP) block and to normalize habituation over successive blocks, whereas inhibiting low frequency rTMS had negligible effects on both. By contrast, in healthy volunteers, inhibiting rTMS reduced first VEP and SSEP amplitude block as well as habituation, while the activating protocol had no effect [42,55]. A longer lasting effect (several weeks) on VEP was induced with 5 consecutive daily sessions of inhibiting rTMS over the visual cortex in healthy subjects, while the effect of activating rTMS in migraineurs lasted only hours or a few days [45]. Recently, Viganò et al. (2013) applied another activating neuromodulation method, anodal tDCS over the visual area in migraineurs, and reported that, similar to 10Hz rTMS, the the 1st VEP block increased in amplitude and habituation normalized [96]. In the second phase of their study, the same authors performed a preventive pilot trial with 2 sessions of anodal tDCS over the visual cortex per week for 8 weeks in 15 migraine patients and found a clear beneficial effect on several clinical endpoints up to an average of 4.8 weeks after the tDCS treatment period [96]. Overall, the neuromodulatory studies indicate that only procedures that enhance cortical excitability are able to normalize the abnormal interictal information processing in migraine.

Further information on the pathophysiology of the interictal dysfunction in migraine was obtained from the more sophisticated studies of the high-frequency oscillations (HFOs) embedded in common somatosensory and visual evoked potentials. Early somatosensory HFOs, reflecting spike activity in thalamo-cortical cholinergic drives, were decreased interictally in migraine and normalized during the attack while late HFOs, reflecting primary cortical activation, were normal [97] or decreased [98]. Moreover, the reduction early HFOs was associated with a worsening in the clinical course of migraine [90]. In a recent study in migraineurs activating rTMS over the sensorimotor cortex was able to increase the interictal low thalamo-cortical drive. This was not the case in healthy volunteers probably because their thalamo-cortical activity was already maximal before the rTMS [97]. This finding supports the hypothesis that deficient habituation in migraine is due to a reduced thalamic control of the activity in sensory cortices i.e. a low pre-activation level. Further evidence for an abnormal thalamic control in the migraine brain intericatlly comes from the analysis of visual HFOs (gamma-band oscillations, GBO) [46]. We demonstrated a significant habituation deficit of the late GBO components in migraineurs relative to healthy subjects, which we interpreted as indicative of a dysfunction in cortical oscillatory networks that could in turn be due to an abnormal thalamic pacemaker rhythmic activity, namely “thalamo-cortical dysrhythmia” [46]. The latter may reconcile the long-lasting controversy between excessive excitation and deficient inhibition in migraine, since a deficient thalamo-cortical drive, i.e. a low level of cortical preactivation, results in dysfunction of both inhibition and excitation. Lower inhibition and preactivation may thus co-exist, since the latter can promote the former via reduction of lateral inhibition [93]. Refined VEP techniques have shown that it is possible to enhance the relative contributions that arise from short- and long- range lateral inhibition between neurons through differential temporal modulation of adjacent regions of radial windmill-dartboard (W-D) or partial-windmill (P-W) visual patterns [99-101]. This may represent a further tool for investigating migraine pathophysiology. According to our recent study, the degree of short-range lateral inhibition in the visual cortex during W-D visual stimulation is more pronounced in migraine patients than in healthy volunteers at the beginning of the stimulus session (1st block). Over successive blocks of recordings, however, it decreases in migraineurs, but remains unchanged in healthy controls. During the migraine attack, short-range lateral inhibition is on the contrary much reduced, but it increases during stimulus repetition. There was no significant between group difference in the P-W amplitude, reflecting long-range lateral inhibition, and its attenuation [88]. These results favour a migraine cycle-dependent imbalance between excitation and inhibition in the visual cortex that results in a heightened cortical response to repeated stimuli, i.e. a lack of habituation. We hypothesized that an interictal hypoactivity of monaminergic pathways may cause a functional disconnection of the thalamus in migraine leading to an abnormal intracortical short-range lateral inhibition, which could contribute to the habituation deficit observed during stimulus repetition. That in migraine the thalamus abnormally controls the cortex via thalamorcortical loops is further underscored by the recent study of the paired associative stimulation (PAS) paradigm, a protocol that uses in humans a design principle very similar to those producing long-term depression (LTD) or potentiation (LTP) in animal studies [102-104]. In migraine, depressing PAS paradoxically increased motor evoked potential amplitudes instead of decreasing them, and enhancing PAS induced only a slight non significant response potentiation [105]. This suggests that impaired long-term associative synaptic plasticity mechanisms characterize migraine without aura patients between attacks. Because we observed, at least in a subgroup of subjects, that the PAS-induced plastic changes were inversely related with thalamocortical activation, as assessed by early somatosensory HFOs, we suggested that the malfunction in PAS-induced effects in migraine might reflect low cortical preactivation, which prevents short-term and longer-term changes in cortical synaptic effectiveness [105].

### Sensitization in migraine

The majority of the studies on the dynamic behavior of peripheral reflexes and evoked cortical responses in migraine have focused on habituation instead of sensitization, probably because it is particularly difficult to assess sensitization by calculating “sliding” averages using successive blocks of few responses.

However, if sensitization, defined as facilitation occurring at the beginning of the stimulus presentation, is impaired in migraine (i.e. enhanced in comparison to habituation), it should be detectable as a higher first block absolute amplitude value of the considered response.

Indirect signs of sensitization were observed during migraine attacks and in chronic migraine patients with or without medication overuse.

It was reported several times that within the 12–24 hours preceding the attack the habituation pattern of reflex and non-noxious evoked potentials normalize. This has been shown with CNV [26,79,80], VEP [50,78,88], visual P300 latency [35], and nociceptive blink reflexes [60].

Pain-related responses may behave in a partially different way. During migraine attacks, the area-under-the-curve of the nociceptive blink reflex R2 component is temporary increased on the affected side in comparison with the non-affected side was observed [106]. Similar results were obtained using another noxious stimulation, the radiant laser CO2: amplitude of the N2–P2 complex at the vertex was increased on the affected side compared to the non-affected side [107-109]. Interestingly, in episodic migraine LEPs did not habituate not only interictally, but also during the attacks, underscoring the different cerebral processing of noxious versus innocuous stimuli. These data could represent the electrophysiological counterpart of central sensitization of cerebral structures belonging to the so-called “pain matrix” that seems to be related to the mechanism of the migraine attack and of the chronification of migraine. As abovementioned, from a physiological point of view, sensitization refers to the plastic changes in neural structures belonging to the “pain matrix” that result in decreased nociceptive thresholds and increased responsiveness to noxious and innocuous peripheral stimuli, and expansion of the receptive fields of CNS nociceptive neurons [14]. As a matter of fact, reduced pain thresholds have been found clinically with quantitative sensory testing immediately before [110] and during [16] a migraine attack, but sometimes even earlier in the interictal period in some [111,112] but not all the studies [110,113-115]. During attacks of migraine, reduced cutaneous pain thresholds on both symptomatic and non-symptomatic sides are accompanied by significantly increased N2-P2 complex of LEP [107] and changes in its dipolar source localization [108]. These abnormalities worsen with the increase in attack frequency [107,108]. In a group of chronic migraine (CM) patients, a trend for an increase in LEP N2-P2 amplitudes [116] and a reorganization of the cortical areas devoted to pain processing [117], was also detected. Moreover, in CM due to medication overuse LEP N2-P2 amplitude still showed reduced habituation after both hand and face stimulation, similarly to the response behavior found with LEPs during interictal and ictal periods [118]. Interestingly, withdrawal from the acute medication overuse normalized the habituation curve [118].

Recently, somatosensory evoked potentials (SSEPs) proved to be ideal for disclosing sensitization (reflected by an increased response amplitude to low numbers of stimuli) and habituation (reflected by a decrease in response amplitude after high numbers of stimuli) in both episodic and chronic forms of migraine. While SSEPs confirmed a lower initial amplitude and late abnormal habituation in migraineurs studied interictally [53,54], a clear-cut sensitization, as reflected by a significant increase in SSEP 1st N20-P25 block amplitude, was found during an attack, followed by a normal habituation [54]. In medication overuse headache (MOH) patients, we managed to record SSEPs in a pain-free state or during mild headaches and found that N20-P25 SSEP amplitude was initially (1st block) greater in MOH patients than in the subgroup of episodic migraineurs studied interictally and healthy controls [54]. The increased SSEP amplitude in MOH was proportional to the duration of headache chronification. We interpreted these results as reflecting reinforcement and perpetuation of central sensitization due to the medication overuse and increased headache frequency [54]. We further noted that the presence of such sensory sensitization depends on the class of drugs overused, since initial SSEP amplitudes were smaller in triptan overusers than in NSAIDs or combined overusers. The abnormalities in cortical responses to somatosensory stimulation seem to be strongly influenced by genetic factors. MOH patients carrying the D/D polymorphic variant of the angiotensin converting enzyme (ACE), that plays a role in neural plasticity and dependence behaviour, showed less SSEP habituation, in proportion with the duration of the overuse headache, and increased sensitization depending on the overused drug compared to I carriers [89].

Sensitization in migraine patients who evolved to chronic daily headache due to medication overuse was also demonstrated with pain-related evoked potentials (PREPs). Ayzenberg and co-workers recorded PREPs after electrical stimulations of cephalic (forehead) and extracephalic (hand dorsum) sites with a nociception-specific electrode. They observed a significant increase in PREP amplitudes both after cephalic and extracephalic stimulations in all patients with MOH irrespective if they overused NDAIDs or triptans. Withdrawal from the acute medication overuse normalized the PREP amplitude [119].

Sensitization phenomena might also manifest themselves at the spinal level. Perrotta and coworkers explored the spinal cord pain processing by studying threshold, area and temporal summation threshold (TST) of the lower limb nociceptive withdrawal reflex in a group of 31 MOH patients before and after acute drug withdrawal. A significantly lower reflex threshold, higher amplitude and lower TST was found in MOH patients before detoxification in comparison with episodic migraine and controls [120]. All these neurophysiological abnormalities tended to improve after a detoxification program [120], which was coupled with an increased activity of the endocannabinoid system [121].

### Habituation in tension-type headache

There are only a few reports about habituation in tension-type headache (TTH).

No habituation deficit was observed with visual evoked or event-related potentials in episodic [41] or chronic TTH patients [34,41]. Episodic TTH sufferers had normal habituation of P300 latency, while P300 amplitude also showed some degree of habituation, although not of statistical significance [122].

Patients affected by chronic TTH showed a normal reducing behavior (habituation) in scalp potentials evoked by CO2 laser stimulation (LEPs) of the hand and facial skin [66]. Mismatch negativity, which is believed to reflect the automatic central processing of a novel stimulus, and P300 habituation were significantly lower in migraine and TTH children than in healthy subjects in one study, where P300 habituation also positively correlated with behavioral symptomatology [123]. In TTH, habituation was also investigated using sympathetic skin responses (SSR), a tool used to evaluate autonomic dysfunction. Ozkul and Ay explored SSR changes with the same stimulus at a constant intensity by four blocks of 20 responses and found that in both episodic migraine without aura and TTH patients there was a lack of habituation compared to normal controls [124]. The electrophysiological similarities between the episodic forms of migraine and TTH support the hypothesis that some patients with TTH might be at the mild end of the migraine spectrum. Since lack of habituation was not always observed in TTH, it seems to be relevant only for a subgroup of patients.

### Sensitization in tension-type headache

In TTH and in cluster headache, like in migraine, the vast majority of neurophysiological studies have only indirectly assessed sensitization, through the measure of the grand-average area under the curve or amplitude of the given test.

The few studies in which the dynamic behavior of responses was analyzed using successive blocks of a few averagings were unable to find clear evidence for sensitization, i.e. for increased amplitude of the first block. This was the case in episodic TTH for VEPs [41], visual P300 [122], LEPs [123] and sympathetic skin responses [124], in chronic TTH for visual P300 [34], and LEPs [66].

By contrast, some indirect evidence for sensitization was found in TTH, chiefly in its chronic form, with nociceptive specific reflexes and evoked potentials.

Normal amplitude, area, latency [125-128] and slower recovery cycle [127] of the blink reflex R2 component was found in chronic TTH. Two separate groups found reduced latencies of the trigemonocervical reflex in patients with chronic TTH [129-131]. Using a nociception-specific electrode lower values of the normalized root mean square and area under the curve of the blink with control subjects [132].

More convincing evidence for central sensitization in CTTH has come from studies of pain sensitivity in pericranial or lower limb tissues. Sandrini et al. (2006) studying the nociceptive lower limb flexion RIII reflex found significantly lower subjective pain thresholds and RIII reflex threshold in chronic TTH than in controls [115]. These findings were associated with a paradoxical facilitation of the RIII reflex response during the cold pressor test, which indicates deficient descending inhibition, an abnormality also found by others [133]. Previous studies have found normal pressure pain thresholds (PPTs) in episodic TTH [134,135]. In chronic TTH instead, PPTs were decreased [15,136,137] especially on the anterior part of the temporalis muscle [15,135-139] and in the upper part of the trapezius muscle [140]. Cathcart et al. (2010) investigated temporal summation, defined as the increase in pain perception to repeated noxious stimulation (indirect measure of sensitization), by an algometer and heterotopic noxious conditioning stimulation (HNCS) in chronic TTH vs. controls. Pain from repeated algometer pressures increased more in the CTTH sufferers compared with healthy controls, both at finger and shoulder, and was less inhibited by conditioned HNCS [141]. Lower pain thresholds in muscle and skin of the cephalic region but not of the extracephalic region with higher rating to suprathreshold single and repetitive (2 Hz) electrical stimulation were reported in patients with chronic TTH than in healthy controls [142].

In a LEP study, the heat pain threshold was similar in chronic TTH patients and controls at the level of both the hand and pericranial skin. The total tenderness scores (TTS) at pericranial sites were higher in TTH patients than in controls. The amplitude of the N2a–P2 LEP complex elicited by stimulation of the pericranial zone was greater in TTH patients than in controls and this was significantly associated with the TTS score [143].

### Habituation in cluster headache and other trigeminal autonomic cephalalgias (TACs)

During the last decades, great advance in the understanding of the cluster headache pathophysiology was made with the modern techniques of functional neuroimaging [144]. Electrophysiological methods contributed to the study of cognitive and nociceptive processes.

Normal cognitive habituation was found in two visual event-related potential studies in cluster headache either during the bout or outside, and in chronic paroxysmal hemicrania [34,145].

Formisano et al. were the first to found abnormal habituation of the blink reflex in a small number of CH patients during the attack, but comparison with control subjects was lacking [146]. Habituation of both the R2 and the R3 blink reflex components are impaired in CH patients on the affected side compared to healthy controls [147]. The lack of habituation in CH patients was even more pronounced than that found in episodic migraine [147]. We recently replicated these results by using the nociception-specific concentric stimulating electrode: R2 reflex area and habituation were reduced on the affected CH side (data published in abstract form [148]). Conversely, Holle et al. (2012) failed to detect altered habituation of the nBR R2 in episodic and chronic CH within or outside a bout. In the latter study, however, the majority of CH patients were taking one or several prophylactic medications at the time of recordings, which may biased the results [149].

### Sensitization in cluster headache and other trigeminal autonomic cephalalgias (TACs)

Classical blink reflex studies did not disclose any sign of sensitization in CH [150,151]. In 10 episodic cluster headache patients within a bout, Lozza et al. (1997) found a significantly faster R2 blink reflex recovery curve on the symptomatic side after paired supraorbital stimuli, probably reflecting an indirect sign of sensitization within the spinal trigeminal nucleus. Furthermore, in the same study the R2 recovery curve was faster on both affected and unaffected sides in CH patients when the supraorbital stimulus was preconditioned by a peripheral stimulation of the index finger. Since naloxone injection transiently reverted this bilateral R2 sensitization, the authors postulated that the faster R2 recovery reflects hypoactivity of reticular nuclei, due to reduced descending opiatergic inhibition [152], a mechanism that was recently supported by functional neuroimaging studies [153,154].

The threshold of the corneal reflex was reduced on the affected side in a mixed group of episodic (during the bout) and chronic CH patients, and normalized in the remission phase [155]. Others were not able to confirm such lateralized abnormalities.

Researchers found that both in- and out-side the bout patients had lower thresholds for pressure pain [156], electric pain and RIII reflex [157] on the affected than on the unaffected side both in episodic (in and outside of a bout) and chronic CH [158]. These signs of sensitization within the nociceptive system were coupled with a phase shift of the normal circadian rhythmic variations in RIII threshold in episodic bouts of CH when compared with the remission period, and with absence of circadian rhythmicity in chronic CH patients [158]. Perrotta et al. (2013) recently studied the functional activity of the descending diffuse noxious inhibitory controls (DNIC) (or conditioned pain modulation system) elicited by a cold pressor test (CPT) in a group of episodic CH patients during active and remission phases. Compared to healthy subjects, the RIII reflex threshold and TST were lower and the R2 area higher during, but not outside of a bout. CH patients during the bout had a significant reduction of TST compared both to controls and to CH patients outside of a bout. Only during the bout but not outside, the CPT had no effect on TST and reflex area [159]. The authors concluded that CH patients have a dysfunction of the supraspinal control of pain that depends on the clinical activity of the disease and leads to facilitation of pain processing predisposing to the CH attacks.

Further evidence for lateralized abnormalities came from the study of Procacci et al. (1989) who found cutaneous and deep hyperalgesia to both mechanical and electrical stimuli with earlier appearance of pain after an ischaemic test in the upper limbs on the affected side of the body in episodic CH patients [160]. By contrast, with quantitative sensory testing, perception of warmth, cold and pressure pain was reduced on the cluster side as compared with the contralateral asymptomatic side in a pooled group of episodic and chronic CH patients [161,162]; warm detection thresholds and thermal sensory limen on the affected side correlated negatively with elapsed time since last attack [162].

We are aware of only one study on sensitization in other trigeminal autonomic cephalalgias. In 12 patients with chronic paroxysmal hemicrania and 12 with hemicrania continua, pain pressure threshold, subjective pain perception after sural nerve stimulation as well as RIII reflex threshold were reduced mostly on the affected side, compared to healthy subjects [163]. Moreover, although there were no abnormalities in the blink reflex, corneal reflex thresholds were significantly reduced on both sides only in chronic paroxysmal hemicrania patients.

### Discussion

**Figure 1:**
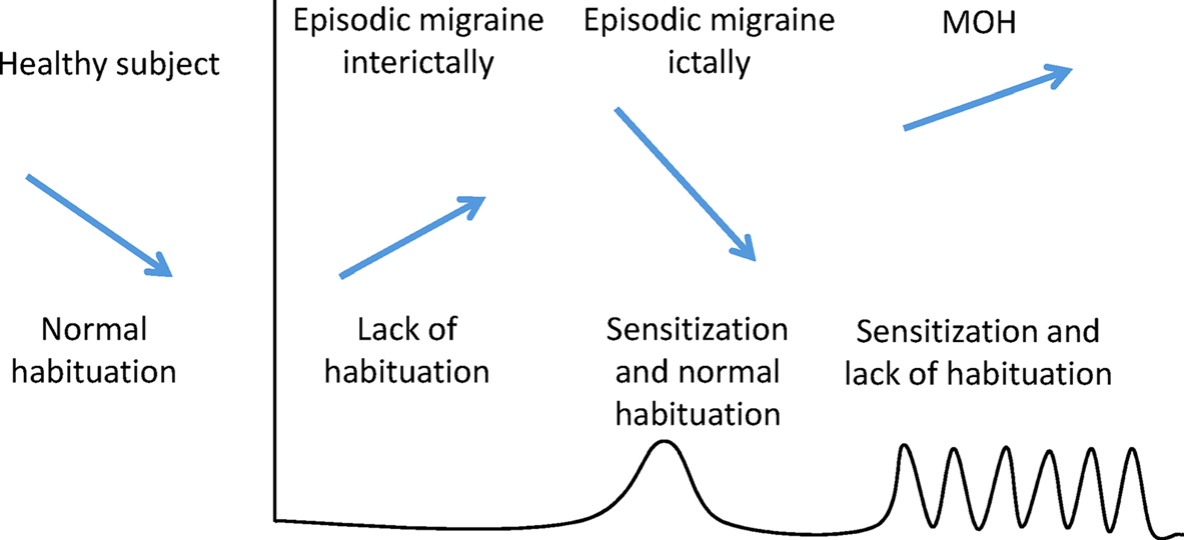
Schematic representation of the changes in habituation and sensitization in an healthy subject and over the migraine cycle (interictal, ictal, and chronic migraine due to medication overuse [MOH]).

Neurophysiological studies have disclosed various abnormalities of spinal, brainstem and cortical responsivity to external innocuous or noxious stimuli in primary headaches. These abnormalities can be summarized as follows:

• Abnormalities of the habituation/sensitization mechanisms were discovered in migraine. In episodic migraine, most published EP studies show two characteristic changes: a lack of habituation on recordings performed between attacks and sensitization during the attack, especially with somatosensory stimuli. The habituation deficit normalizes during attacks, whereas sensitizations vanishes between attacks, but in the immediate pre-ictal phase both sensitization and deficient habituation may variably co-exist in response to non-noxious and pain stimuli. In patients who developed MOH the cortical response pattern could be locked in a pre-ictal state associating both initial sensitization and late deficient habituation, which contrasts with episodic migraine where these cortical states alternate (Figure 1). Recent works suggest that an abnormal rhythmic activity between thalamus and cortex, namely thalamocortical dysrhythmia, may be the pathophysiological mechanism subtending abnormal information processing in migraine.

• In tension-type headache, available studies are limited. Some, though only in subgroups of patients, found some evidence of deficient habituation, chiefly with cognitive potentials (mismatch negativity and P300) and sympathetic skin responses. By contrast, using grand-average responses indirect evidence for sensitization has been disclosed in chronic TTH with nociceptive specific reflexes and evoked potentials. These studies provide evidence for generalized increased sensitivity to pain (lower thresholds and increased pain ratings) and a dysfunction in supraspinal conditioned pain modulation in CTTH, which may contribute to the development and/or maintenance of central sensitization in this disorder.

• The deficient habituation of the blink reflex found in episodic CH patients during the bout suggests that interictal migraine and cluster headache probably share some pathophysiological mechanisms. However, the more pronounced habituation deficit found in the CH with respect to the migraine group suggests that additional dysfunctional neurobiological factors are at work in CH patients. Only during the bout but not outside, a sensitization of pain processing was observed. Several possible non-mutually exclusive causes could be responsible for this: i) dysfunctioning descending aminergic, especially dopaminergic, control [164,165], ii) malfunctioning hypothalamo-trigeminal control [166], and iii) altered descending opiatergic pain control system [153,154]. Given that CH patients had no habituation deficit of event-related potentials [34,145], it is likely that the pathogenic factors involved in CH produce functional changes at the level of the trigeminal system but not at cortical level. Altogether, these data indicate that in cluster headache lateralised abnormalities may occur throughout the body, probably because of sensitization in the central nervous system and activation of nociceptive reflexes.

## Conclusions

In migraine research, progress will largely depend on a better understanding of the mechanisms underlying the habituation deficit, its variations with the migraine cycle and its relation with changes in thalamo-cortical rhythms and brain stem-(thalamo-)cortical aminergic pathways. Future studies will have to determine whether there is an interaction between abnormal sensory processing and metabolic abnormalities, for instance decreased brain ATP content.

It will also be of importance to gather more data on the geno- phenotype correlations in migraine and in the other primary headaches. Such genotype/phenotype correlations could help to tailor treatment to the individual patient depending on his genetic profile [89].

In cluster headache, future electrophysiological works should try to understand the role of the descending monoamine and opioid systems in the mechanism of sensitization and lateralization of pain. Moreover, they may help to unravel the mechanisms that periodically ignite the cluster period and the culprits for the transformation of episodic into chronic CH.

Finally, better characterizing headache patients from a neurophysiological point of view will allow to optimize the protocols of the minimally invasive techniques and non-invasive neurostimulation methods, and to improve their therapeutic efficacy [96,167,168].

## Abbreviations

BR: Blink reflex; CH: Cluster headache; CM: Chronic migraine; CNV: Contingent negative variation; CPT: Cold pressor test; CTTH: Chronic tension-type headache; EP: Evoked potential; GBOs: Gamma-band oscillations; HFOs: High-frequency oscillations; HNCS: Heterotopic noxious conditioning stimulation; LEP: Laser evoked potential; MOH: Medication overuse headache; OFC: Orbitofrontal cortex; PAS: Paired associative stimulation; PREPs: pain-related evoked potentials; PPTs: Pressure pain thresholds; P-W: Partial-windmill; RR: Reflex response; rTMS: repetitive Transcranial Magnetic Stimulations; SSEP: Somatosensory evoked potential; SSR: Sympathetic skin responses; tDCS: transcranial Direct Current Stimulations; TST: Temporal summation threshold; TTH: Tension-type headache; TTS: Total tenderness score; W-D: Windmill-dartboard.

## Competing interests

The authors declare that they have no competing interests.

## Authors’ contributions

GC made substantial contributions to review the literature as well as in drafting the manuscript. CDL, JS, and FP were implied in drafting the manuscript. All authors read and approved the final manuscript.
